# Prevalence of tick borne encephalitis virus in tick nymphs in relation to climatic factors on the southern coast of Norway

**DOI:** 10.1186/1756-3305-5-177

**Published:** 2012-08-22

**Authors:** Ashild Andreassen, Solveig Jore, Piotr Cuber, Susanne Dudman, Torstein Tengs, Ketil Isaksen, Hans Olav Hygen, Hildegunn Viljugrein, Gabriel Ånestad, Preben Ottesen, Kirsti Vainio

**Affiliations:** 1 Division of Infectious Disease Control, Department of Virology, Norwegian Institute of Public Health, P. O. Box 4404, Nydalen, 0403, Oslo, Norway; 2 Department of Pest Control, Norwegian Institute of Public Health, P. O. Box 4404, Nydalen, 0403, Oslo, Norway; 3 Norwegian Veterinary Institute, Ullevålsveien 68, P.O.Box 750, centrum, 0106, Oslo, Norway; 4 School of Pharmacy, Department of Parasitology, Medical University of Silesia in Katowice, 41-218, Sosnowiec, Ul. Jedności 8, Poland; 5 The Norwegian Meteorological Institute, Oslo, Norway; 6 Centre for Ecological and Evolutionary Synthesis (CEES), Department of Biology, University of Oslo, Oslo, Norway

**Keywords:** Prevalence, TBEV, Estimated pooled prevalence, MIR, Climate, Pooled sampling, *Ixodes ricinus*

## Abstract

**Background:**

Tick-borne encephalitis (TBE) is among the most important vector borne diseases of humans in Europe and is currently identified as a major health problem in many countries. TBE endemic zones have expanded over the past two decades, as well as the number of reported cases within endemic areas. Multiple factors are ascribed for the increased incidence of TBE, including climatic change. The number of TBE cases has also increased in Norway over the past decade, and the human cases cluster along the southern coast of Norway. In Norway the distribution and prevalence of TBE virus (TBEV) in tick populations is largely unknown. The objectives of this study were to estimate the TBEV prevalence in *Ixodes ricinus* from seven locations and to assess the relationship between the TBEV prevalence and site-specific climatic variables.

**Methods:**

A total of 5630 questing nymphs were collected and analyzed in pools of ten. All pools were screened with an in-house real-time RT-PCR, and the positive pools were pyrosequenced. Two methods, minimum infection rate (MIR) and a frequentist method (EPP) for pooled prevalence estimations were calculated and compared. Climatic data were descriptively compared to the corresponding EPP of each location in order to explain variations in TBEV prevalence.

**Results:**

The seven foci of TBEV had an estimated overall prevalence (EPP) in pools of nymphs combined, of 0.53% with 95% CI (0.35–0.75), with point prevalence ranging between 0.11%–1.22%. The sites with the highest point prevalences were within the municipalities which had the highest numbers of registered TBE cases. The results indicate that the location with highest point prevalence had the highest relative mean humidity and lowest mean saturation deficit and vice versa for the lowest EPP.

**Conclusion:**

Our study confirms the existence of TBEV endemic foci in Norway. These results are of importance to increase the awareness of TBEV infections in Norway and could be used for public information and recommendations of TBE vaccination. EPP is the method of choice for pooled prevalence calculations, since it provides estimated prevalences with confidence intervals. Our findings emphasise the possible importance of microclimatic conditions regarding the TBEV prevalence in ticks.

## Background

Tick-borne encephalitis virus (TBEV) is the causative agent of one of the most important flaviviral infections in Europe and Asia [1]. This zoonotic disease is endemic in Central and Northern Europe as well as Siberia and Japan [2]. TBEV is a neurotropic virus that may cause fatal meningitis, encephalitis and/or radiculitis [3]. Three main TBEV subtypes, which are closely related both genetically and antigenically, have been described according to their main distribution area: TBEV-European (TBEV-Eu), TBEV- Siberian (TBEV-Sib) and TBEV- Far Eastern (TBEV-Fe) [4]. The co-circulation of the different TBEV subtypes is reported in some regions such as the Baltic States and Finland [5]. The disease caused by the different subtypes of TBEV varies in severity, though the TBEV-Eu case fatality rate rarely exceeds 1% [6]. Humans are infected via tick bites or much more rarely by the consumption of unpasteurized milk or dairy products from sheep, goats and cows [7,8]. Ticks act as both a vector and reservoir of TBEV and all three subtypes can be transmitted by ticks. The main vector in the Central, Eastern and Western Europe is *Ixodes ricinus* (L., 1758) while *I. persulcatus,* Schulze, 1930 is the main vector in Asia and Eastern Europe [2,9]. TBEV has also been detected in *Dermacentor* spp. [10]. Humans are accidental viral hosts, with the main hosts being small rodents [11].

TBEV occurs in discrete foci of variable sizes within a tick distribution area [12] because climatic and ecological conditions determine the TBEV transmission efficiency. The ratio of co-feeding ticks on hosts seems to be one of the factors that separate a TBEV focus from a non-TBEV focus [13]. Rapid warming in springtime expedites nymph and larval co-feeding on rodent hosts [14], and local microclimatic conditions can act to constrain the degree of co-feeding [15].

TBE has been a growing public health concern in Europe and other parts of the world over the last decades [16]. In Norway and a few other European countries, TBE is regarded as a minor public health problem [16,17]. This is in contrast to the majority of European countries however, including Switzerland and Sweden, where TBE is considered to be of significant importance [18-22]. Detailed knowledge of the distribution and prevalence of vector-borne viruses is required in order to provide appropriate vaccine recommendations and to carry out hazard assessments. The incidence of TBE in the European countries increased between 1974 and 2003 with the exception of Austria, where a very high rate of vaccination occurs [22,23]. Over the last decades the geographic range of TBEV and its disease occurrence has increased [9,24]. These increasing trends are likely due to a complex combination of expanding tick populations as a result of increasing abundance of Roe deer and other deer species as tick hosts [25,26], climatic factors [27,28], social and behavioural changes [29,30] changes in land use and leisure activities [29], in addition to increased recognition and reporting of TBE cases [16,31]. Nevertheless, the virus transmission cycle is very vulnerable to disruption if high temperatures and low humidity prevail. Climatic change alone does not seem to account for the increased incidence of TBE in the Baltic countries [14].

The first description of a tick-borne encephalitis-like disease dates back to the 18^th^ century, in which it is described in Scandinavian church records [2]. In the Scandinavian countries, the first official reports of TBE from Sweden, Finland, Denmark and Norway were in 1954, 1956, 1963 (Bornholm) and 1997, respectively [17].

Only one study has been published on TBEV in the tick populations in endemic areas of Norway, thus knowledge regarding the distribution and prevalence of TBEV is limited [17]. Skarpaas et al. (2006) estimated the prevalence in ticks to be 0.2–0.3% at one location (Tromøya) on the southern coast of Norway [17]. TBE is a notifiable disease in Norway and all the human cases are reported by physicians to the Norwegian Surveillance System for Communicable Diseases (MSIS) [32]. According to MSIS, the annual number of reported cases of TBE in Norway has increased from 2 to 14 cases during the last ten years [32]. TBEV risk areas can be defined by extrapolating data on the regions with TBE cases and estimating virus prevalence in ticks. The data on TBEV prevalences is important for the implementation of relevant health authority measures such as vaccination guidelines. The objectives of this study were to determine TBEV prevalence in ticks collected from seven locations and to assess how the estimated TBEV prevalences may relate to site-specific climatic factors.

## Methods

### Collection and storage of ticks

**Figure 1:**
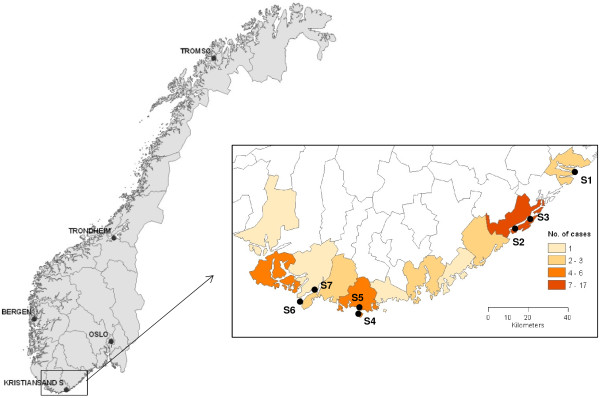
**Tick sampling locations at the southern coast of Norway.** The insert with various colouring of the regions describes the number of reported cases with TBE infections according to MSIS in the period 1998–2010.

**Figure 2:**
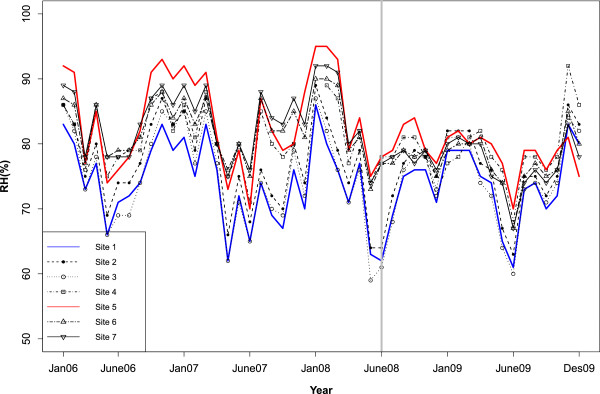
**Relative humidity (RH): Monthly variation in the mean relative humidity (%) from 2006 to 2009 for the seven sampling sites.** The grey line indicates the RH one year before sampling.

**Figure 3:**
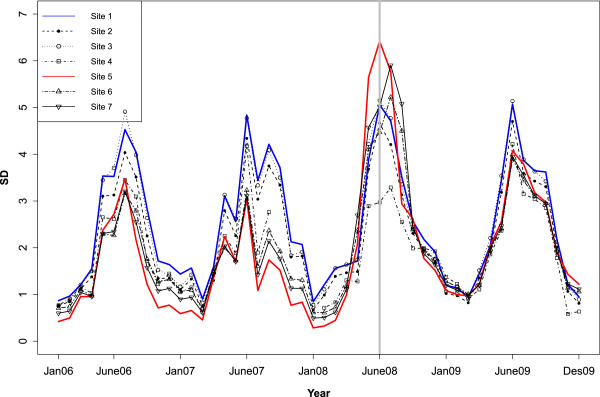
**Saturation deficit (SD): Monthly variation in the mean saturation deficit from 2006 to 2009 for the seven sampling sites.** The grey line indicates the SD one year before sampling.

Ticks were collected at seven locations along the southern coast of Norway (Figure 1). The various locations were chosen according to registrations of human TBE cases between 1998 and 2010 in MSIS [32] and from information by local general practitioners and the public (Additional file 1: Appendix). The distance between the edges of locations where the human cases were reported was only 146 km and all sites in this study are located near the coastline and have a typical coastal climate. The names and descriptions of the sample site localities are given in Table 1. Roe deer were present at all sites, and in addition grazing sheep were present at site S4. Ticks were sampled daily from the 15^th^ to 18^th^ of June 2009 (Figures 2 and 3). Questing ticks were collected by “the woollen flannel cloth dragging method” [33]. The cloths were 1.1 m by 0.65 m. The flannel cloth was attached to a bamboo cane by adhesive tape at each end of the cloth. Dragging was performed on the ground or through the vegetation. Stops were made after a couple of sweeps, the cloth was inspected and all ticks were removed by forceps and counted before putting them into an Eppendorf tube. Collected ticks were stored on crushed ice during transportation to the laboratory. Ticks were stored at −18°C for two days and then transferred to −70°C. Later the ticks were randomly sorted per site in tubes on blocks of frozen CO_2_ and the nymphs were selected into pools of ten. The whole material consisted of 5630 nymphs. Larvae and adults were not included.

**Table 1:** Sampling sites with global position coordinates, vegetation and climatic factors

**Site**	**Location**	**UTM coordinates**	**Growing season (mean)**	**Spring- days**	**Autumn- days**	**RH Mean (%) [min-max]**	**SD mean [min-max]**	**Total precipitation mean (mm) [min-max]**	**Temp mean (°C) [min-max]**	**Temp min (°C) [min-max]**	**Temp max (°C) [min-max]**
S 1	Risør, Dalen	32 V051068-	199	13	6	74	2,49	131,99	8,35	3,62	12,74
		26504532				[61–68]	[0.85–5.07]	[25.4–294.3]	[−2.23–18.74]	[−9.9–15.4]	[2.7–23.1]
S 2	Arendal, Tromøya	32 V049237-	199	6	5	77	2,21	126,41	8,53	3,97	12,68
		86478782				[63–89]	[0.67–4.70]	[21.1–290.1]	[−1.88–18.77]	[−9.2–15.2]	[3.0–22.3]
S 3	Arendal,	32 V049461-	201	14	6	75	2,44	126,50	8,48	3,88	12,68
	Skarestrand	16482379				[59–87]	[0.75–5.15]	[21.7–284.2]	[−2.04–18.84]	[−9.6–15.4]	[3,0–22.5]
S 4	Mandal,	32 V041309-	200	8	5	80	1,90	160,59	8,71	4,38	12,90
	Skjernøya	06427922				[68–92]	[0.59–4.22]	[27.2–357.2]	[−1.07–18.54]	[−8.8–15.0]	[4.2–22.5]
S 5	Mandal,	32 V041186-	200	7	4	82	1,89	160,59	8,62	4,24	12,88
	Skjernøysundkilen	16430610				[70–95]	[0.29–6.42]	[27.2–357.2]	[−1.20–18.61]	[−9.6–14.9]	[4.1–22.8]
S 6	Lyngdal, Vasstøyl	32 V038251-	202	7	4	81	1,97	179,99	8,60	4,41	12,63
		76435547				[67–90]	[0.61–5.22]	[28.5–396.2]	[−0.91–17.51]	[−7.6–14.2]	[4.0–22.5]
S 7	Lyngdal, Kvalsvik	32 V038260-	206	7	5	81	1,97	192,33	8,49	4,22	12,63
		96441024				[67–92]	[0.49–5.90]	[27.4–422.9]	[−1.09–17.68]	[−8.0–14.3]	[4.0–22.7]

Spring days: number of consecutive days where mean daily temperatures were between 5–8°C in the spring (3 days smoothing).

Autumn days: the number of consecutive days where mean daily temperatures were between 8–5°C in the autumn (3 days smoothing).

Climatic data: is means of daily mean measurements for all 365 days in each year.

### Nucleic acid isolation from nymphs

Each pool of 10 nymphs was homogenized in RTL buffer (QIAGEN Inc., Valencia, CA, USA) with MK-28 steel beads in a Precellyse® 24 Homogenizer 5500 rpm for 20 seconds (MO BIO Laboratories, Inc, Carlsbad, CA, USA). Total RNA was extracted by RNeasy mini kit from the homogenate according to manufacturer’s instruction (QIAGEN Inc., Valencia, CA, USA). RNA was eluted in 40 μl Tris–HCl buffer (1 mM pH 8.0). The quantity (100 ng/μl) and purity of the RNA was determined for ten randomly chosen pools using a NanoDrop ND-1000 Spectrophotometer (Thermo Scientific, Waltham, MA).

### Reverse Transcription (RT)

Five μl total RNA (500 ng) eluted from each nymph pool was reverse transcribed to cDNA using the High Capacity cDNA Reverse Transcription System kit with RNase Inhibitor (Applied Biosystems, Foster city, CA, USA) and random primers according to the manufacturer’s instructions. TBEV (50 000 virus particles/μl of RNA) isolated from a TBE positive case (“Soukupa”) was used in 10^-3^–10^-7^ dilution as standard in each PCR run (kindly provided by Dr. Christian Beuret, Spiez lab, Spiez, Switzerland). cDNA from each pool was either analysed by PCR the day after RT or stored at −80°C until further analysis.

### TBEV PCR assays

**Figure 4:**
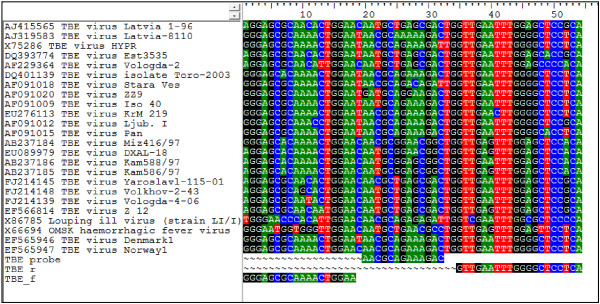
**Alignment of representative TBEV sequences covering the target locus for the real-time PCR assay.** Probe and primers were designed to match Danish and Norwegian strains of the virus, respectively EF565947 and EF565946.

Detection of TBEV in nymph pools was performed using an in-house real-time RT-PCR amplifying a 54 bp fragment of the 3’end of the TBEV E-gene (nt 1662–1715) (Figure 4). The real-time RT-PCR assay was designed for detection of the TBEV strains isolated from a Norwegian and a Danish patient [17]. Probe and primers were designed using the software PrimerExpress (version 2.0.0; Life Technologies Corporation, Carlsbad, California, USA). All positive pools were further analyzed by pyrosequencing. Primers and probes are listed in Table 2.

**Table 2:** Primers and probes used for real-time detection of TBEV*

**Primer name**	**Sequence (5’ → 3’)**	**Genom position**	**GenBank accession No.**
*TBE 320F*	*GGGAGCGCAAAACTGGAA*	1662–1680	U27495
TBE 373R	TGAGGAGCCCCAAATTCAAC	1696–1715	U27495
TBE 339 probe	(FAM)-AACGCAGAAAGAC-(BHQ1)	1681–1693	U27495

*Designed by T. Tengs at the Norwegian veterinary institute.

The real-time PCR was as follows: Master Mix: 1 X “in-house” buffer, 5 mM MgCl_2_, 0.2 mM dUTP, 0.25 μM Forward TBEV 320 F, 0.25 μM Revers TBEV 373 R Biotin labelled, 0.3 μM TBEV probe 339, 0.19 Units Pt-Taq (Invitrogen Life Technology, Inc., Carlsbad, CA, USA), RNase free water (Sigma) up to 22 μl. The real-time PCR was run with a total volume of 25 μl including 3 μl cDNA on the Rotogen 6000 (QIAGEN, Germany) with the following conditions, 1 cycle 94°C 2 minutes, 45 cycles: 94°C 15 seconds, 60°C 45 seconds, 72°C 30 seconds. In each PCR run the standard (see above) was included in order to determine the sensitivity of the PCR method (data not shown). The standard was used as a positive control and water as a negative in each PCR run.

### Pyrosequencing

All real-time PCR positive pools were further analysed by Pyrosequencing according to the manufacturer’s manual for SQA analysis directly after real-time PCR in a BioTage (Pyromark ID) System (QIAGEN, Germany). Real-time PCR was carried out with a biotinylated (bio) TBE 373 R primer (Table 2) to enable efficient preparation of a single strand using streptavidin-coated sepharose beads. For the sequencing reaction 0.44 μM of the TBEV 320 F primer (Table 2) was supplemented, and the reaction was performed using the Pyrogold SQA reagents (QIAGEN, Germany) according to the manufacturer’s instructions. The sequences obtained from the PCR positive pools were compared with the standard.

### Data analyses

#### Sample size calculations

The required sample size (m) to estimate the expected prevalence with a desired precision was calculated as described by Daniel (1999) [34]:

(1) $${\text{m}}_{\left(\text{perfect test}\right)}={\left(\text{Z}\left(1-\text{p}\right)/\left(\text{e}\cdot \text{k}\right)\right)}^{2}\left({\left(1-\text{p}\right)}^{\text{k}}-1\right)$$

where:

p = expected prevalence,

k = pool size

e = the accepted error (desire precision) and

Z = the standardised normal variety corresponding to the desired of confidence

A pool size of ten nymphs was chosen based on the formula above and recommendations of Ebert et al. (2010) [35].

With an expected TBEV prevalence of about 0.3%, based on the previous study of TBEV prevalence in ticks [17], 730 ticks have to be analysed to be able to estimate prevalence with a 95% confidence limit and an accuracy of +/− 0.4.

### Pooled prevalence calculations

The rationale and statistical methods for estimating infection rates from pooled sampling has been described elsewhere [35,36]. Two different methods for pooled prevalence estimation were chosen and compared;

a) Minimum Infection Rate (MIR):

(2) $$\text{MIR}=\left(\text{x}/\left(\text{m}\text{k}\right)\right)100\%$$

where:

This is the most basic method for analyses of pooled samples where one assumes that a positive pool is infected by a single individual [36-38]. The assumption that each pool has only one positive cannot be proven and the method is too imprecise unless the prevalence is extremely low, e.g. <0.1% [36].

k = pool size,

m = the number of pools tested

x = the number of positive pools

b) The frequentist method to calculate maximum-likelihood estimates of pooled prevalence and confidence limits (EPP):

Pooled prevalence was calculated by using an online pooled prevalence calculator of the Epitools epidemiological calculators [39]. The method uses the frequentist approach to estimate prevalence and confidence limits, assuming a fixed pool size and perfect (100%) test sensitivity and specificity. Since the assumed prevalence is close to zero, the method (Method 3 from Crowling and co-workers (1999) [36]) with exact confidence intervals was used.

Prevalence was estimated as:

(3) $$\text{p}=1-{\left(1-\text{x}/\text{m}\right)}^{1/\text{k}}$$

and the standard error (SE) is estimated as the square root of the variance, given by:

(4) $$\text{Var(p})=\left(\text{x}/\text{m}\right){\left(1-\text{x}/\text{m}\right)}^{2/\left(\text{k}-1\right)}/\left(\text{m}{\text{k}}^{2}\right)$$

where:

Exact confidence limits are estimated by calculating the corresponding binomial confidence limits for the proportion of positive pools and then transforming these back to individual-level prevalence values using the equation for estimating prevalence.

p = estimated prevalence,

k = pool size,

m = the number of pools tested

x = the number of positive pools.

### Climatic data

Climatic data were obtained from the Norwegian Meteorological Institute [40] and compiled over the period 2006–2009. The weather stations in this study were carefully chosen based on the knowledge of local micro climate conditions and that it had a close relation to the sampling sites. Depending on weather elements and time resolution, different methods were chosen to evaluate the local climate at the seven locations presented in this study:

The modelled grids (1 km² × 1 km²) of daily air temperature (24-hour mean temperature) based on observations at approximately 260 weather stations in Norway [41,42] were used to calculate the growing season (number of consecutive days where mean daily temperatures were above 5°C) and number of days having daily mean temperature between 5 and 8°C in spring and between 8 and 5°C in autumn as suggested by Lindgren and Gustafson [43].

The rate of spring warming measured by a linear regression of temperature against time (ratio) has been used to explain the distribution of TBEV foci. In order to assess whether or not the climate at our sampling locations were consistent with climate at predicted TBEV foci, we estimated the rate of spring temperature increase (Spring warming rate) from February to April 2006–2009 as described by Randolph and Sumilo (2007) [44].

Daily measures of mean, maximum and minimum air temperatures (°C), mean relative humidity (%) and total precipitation were obtained from a manually weighted interpolation of data from seven nearby weather stations. To investigate general trends and differences between sites, weekly, monthly and annual means were calculated for all these parameters. Data from air temperature and relative humidity (RH) were used to calculate saturation deficit (SD) according to Randolph and Storey (1999) [45]. SD reflects both temperature and RH, and gives a measure of the drying power of the atmosphere; RH reflects the amount of water vapour in the air relative to the total amount of moisture that the air can contain. SD and RH were descriptively compared within and between each location, and with regard to the corresponding pooled prevalence of TBEV for each location.

All mapping was performed in ESRI ArcGIS 9.2 (ESRI, Redlands, CA,USA) and analyses of climatic variables were performed in R version 2.6.2 [46].

### Statistics

All statistical significance was assessed at the 0.05 level and hypothesis tests were two-tailed. Descriptive statistics were performed by Pearson Chi-Square test using PASW 17, version 17.02 (SPSS Inc., Chicago, IL, USA).

The Relative Risk (RR) with confidence interval (CI) using likelihood ratio tests was analyzed by the Egret software [47].

## Results

A total of 563 pools with 10 nymphs in each pool were analyzed from seven locations (S1-S7) (Figure 1). Seven hundred and thirty nymphs or more were collected from all sampling locations, except at S3, where we managed to collect only 620 nymphs.

As shown in Figure 1, the highest number of TBE cases has been registered in Arendal. The number of analyzed pools ranged from 62 (S3) to 94 (S7) between the locations (Table 3).

**Table 3:** Estimated pooled prevalence of positive pools: - by frequentist method (EPP) and minimum infection rate (MIR)

**Site**	Number of pools*	**Number of positive pools detected by Real-time PCR**	**Estimated pooled-prevalence by frequentist method on Real-time PCR (95% CI)**	**Estimated pooled prevalence by Minimum infection rate level (MIR) (%)**
S1	90^a^	1	0.11 (0–0.62)	0.11
S2	73	4	0.56 (0.28–1.43)	0.55
S3	62	4	0.66 (0.018–1.69)	0.65
S4	74	5	0.70 (0.23–1.62)	0.68
S5	78^a,b^	9	1.22 (0.56–2.30)	1.15
S6	92	4	0.44 (0.12–1.13)	0.43
S7	94^b^	2	0.21 (0.03–0.77)	0.21

* All pools consist of 10 nymphs.

^a^ site S1 is statistically significant different from site S5; p = 0.006.

^b^ Site S7 is statistically significant different from site S5; p = 0.014.

The estimated prevalence of TBEV was calculated both by the MIR and frequentist methods (EPP) (Table 3). The seven foci of TBEV had an estimated overall prevalence (EPP) in pools of ticks combined, of 0.53% with a 95% CI (0.35–0.75), but ranged from 0.11% to1.22% for the various locations (Table 3). The highest point prevalences were found in Mandal (S4, 0.70% and S5, 1.22%), followed by Arendal (S2, 0.56% and S3, 0.66%). The estimated prevalence in site S5 (1.22%) showed higher statistically significance than site S1 (0.11%) (p = 0.006) (Table 3, Additional file 2: Figure S1), which is in accordance with the relative risk (RR) between S5 and S1 of 10.4 and CI (1.4–8.4) (p = 0.026) (Additional file 3: Table S1). The estimated prevalence at S5 was also significantly higher than S7 (0.21%) (p = 0.014) (Table 3). All other sites had overlapping prevalence confidence intervals and there were no statistically significant differences between these sites.

All real-time RT-PCR positive pools were detected at high Ct values (ranging from 26.4 to 42.4; Median 40.4). Only one pool had a Ct value of 26.4, which was comparable to the Ct value obtained from the undiluted standard. All real-time RT-PCR positive pools were confirmed by pyrosequencing. In total 29 positive pools were detected by real-time PCR, whereas 17 were verified by pyrosequencing (data not shown). The sequence similarity between the standard and the positive pools varied between 70–100%, whereas the different dilutions of the standard (from 10^-3^ to 10^-6^) showed 100% sequence similarity in each run. The lowest dilution of the standard (10^-7^) was PCR negative in each run.

### Climatic data

The climatic variables used in this study displayed spatiotemporal variation (Table 1). Estimated uncertainties for actual temperature in winter and spring months are +/− 1.5°C, while the uncertainty in the summer months typically is within +/− 2°C. The location with the highest EPP had also the highest mean RH and lowest mean SD, whilst the location with the lowest EPP had the lowest mean RH and highest mean SD (Table 1, Figure 2 and 3). The weekly rate of spring temperature increase from February to April (2006–2009) was estimated to be 9.5 for the seven sites (measured by a linear regression of temperature against time). This varies from 7.5 to 9.5 when estimated for each of the seven sites separately. There were no significant differences among the TBEV positive site-specific rates of spring warming (testing the interaction of site against week: p-values ≥0.3). The annual mean January minimum temperature was −3.4°C varying from −2.6 to −4.4°C in the period from 2006 to 2009.

## Discussion

This is the first extensive survey of TBEV prevalences in *Ixodes ricinus* in Norway, and our study confirms the existence of TBEV endemic foci in Norway. The seven localities tested for TBEV (Figure 1) had an estimated overall prevalence rate of 0.53%, which is in accordance with a Swiss study reporting a mean prevalence of 0.46% in endemic areas [48]. The average prevalence in foci of endemic areas in Europe ranges from 0.1–5% [49]. TBEV prevalence point estimates range from 0.11%–1.22% for the various locations in this study and a significantly higher EPP was found at S5 (1.22%) compared to S1 (0.11%) and S7 (0.21%) (Table 3). Sites S3 (0.66%), S4 (0.70%) and S5 exhibit the highest point estimates of prevalence, and are located within the municipalities that have the highest number of TBE cases reported in Norway [32].

Previous studies have proposed that TBEV is distributed in a patchwork pattern, which is probably due to climatic conditions, virus prevalence, vector and host relationship and other factors [13].

The prevalence of TBEV in ticks is a suitable marker for TBE risk analysis in natural foci [50,51], but cannot be directly translated into a risk for the population. The risk of human infection is the product of the hazard (number of infected ticks, which is a product of tick abundance and pathogen infection prevalence) and contact rate between the infected ticks and humans [52]. As a zoonotic agent, TBEVs circulates widely in wildlife and the appearance of the virus is likely to be far more widespread than revealed by human cases [44]. The virus primarily circulates in rodents without human cases being reported in the areas until either the hazard or the contact rate increase above a certain variable level [52]. The distribution of TBE cases is therefore shaped by the distribution of the human population and the behaviour that favours human-tick contact. Due to the focal distribution of TBEV, the estimated prevalence cannot be generalized to reflect the true prevalence in the study region, whereas the prevalence of infected ticks within risk areas has also been reported to vary considerably between sites [9,53]. In Norway the distribution and prevalence of TBE Virus (TBEV) in tick populations is largely unknown. Our findings are important to increase the awareness of TBEV risk areas in Norway, while also being useful for advising the public about TBE infections and its prevention together with vaccine recommendations.

The screening of pooled samples is complicated since it is impossible to determine if a positive result is due to one or more infected ticks. The simplest method to estimate pooled prevalence is the calculation of Minimum Infection Rate (MIR); which assumes that a positive pool only contains a single infected tick and provides an estimate of the average minimum infection prevalence [35,54]. This method is inaccurate and might underestimate the actual level of infection and gives only point prevalence estimates with no information about the uncertainty of the estimate. The Frequentist methods (EPP) calculate maximum-likelihood estimates of true prevalence and confidence limits [36], although the difference between the point prevalence estimations of MIR and EPP in our study is negligible due to the sites having a low prevalence level. However, EPP provides a measurement of the uncertainty contained in the confidence interval associated with the apparent prevalence estimations (Table 3).

Estimating prevalence is subject to errors influenced by the sampling process. This source of error is influenced by both the number of ticks examined and by the methods used to examine them. More than 730 nymphs were obtained from each site in our study, with the exception of the location S3. This allows for a good estimation of prevalence with a high confidence limit and a high precision (Table 3). When prevalence is <10%, pooled testing is comparable to or better than individual testing. This is assuming that the sensitivity of the pool test is equal to the test used for individuals [55,56]. The pool size will obviously influence the variance of a prevalence estimate, and the effect of reducing the pool size depends primarily on the true prevalence in the population investigated. If the prevalence is approximately 0.1%, the standard deviation will be small, so even up to a pool size of 30, i.e. a smaller pool size would not improve the accuracy of the estimates, while if the prevalence is 0.3 an effect will already be seen at a pool size of 8–10 [35]. The effect of having smaller pool sizes is therefore most prominent at the higher prevalence levels, and reducing the pool size would not eliminate the inherent problem with the MIR method. For instance, with a pool size of 10, and assuming a 10% prevalence it is possible that one individual in each pool could be positive, which would result in an estimated prevalence of 100%.

Many papers on TBEV in ticks are based on MIR as a measure of the prevalence of virus infected ticks. A variable number of ticks in each pool, as well as a mix of adults, larvae and nymphs makes it difficult to compare prevalence studies between countries or regions within a given country [17,57-59]. The current study was performed on a large number of nymphs from each site calculating both EPP and MIR, but using EPP as the method of choice. This study used a fixed number of nymphs in each pool, and only nymphs were included. Using this method ensures that similar amounts of nucleic acid were isolated from each pool, thereby resulting in a reliable EPP when comparing TBEV presence and absence. In our study, high Ct values close to the detection limit of the RT-PCR indicated that only a few nymphs in each pool carried the TBEV.

Tick-borne flaviviruses are known to occur at a relatively consistent, low prevalence in tick populations [60]. The mechanism that leads to a stable perpetuation of tick-borne flavivirus in nature has not been fully researched and needs further study [61]. The key to TBEV maintenance was proposed by Labuda and Randolph (1999) [62] to be the non-viraemic transmission of TBEV between infected nymphs and uninfected larvae when co-feeding on rodents. Co-feeding has been shown to be favoured in the spring, when temperatures rise rapidly and exceed 10°C, with the rate of spring warming being used to explain the distribution of TBEV foci. The rate of spring warming together with the mean January minimum temperature, estimated in our study, is in line with Randolph and Sumilo’s (2007) results for TBEV-presence sites [44]. Lindgren and Gustafson found that an increase in TBEV prevalence was significantly related to a temperature range between 5 and 8°C [43]. Randolph and Sumilo used the slope of the 10-day mean Land Surface Temperature (LST) obtained from satellites to calculate the number of days corresponding to a daily mean temperature between 7 and 10°C in spring [44]. LST is regarded as the Earth’s skin temperature; however, the land surface is far from being a skin or a homogenous two-dimensional entity: it is composed of different materials with various geometries both of which complicate LST estimation [63]. In our study, LST data were not suited due to the great variability in surface types within 1 × 1 km in the study areas, corresponding to the pixel resolution in the LST dataset. In addition, there are only minor differences between the slopes of the increase in maximum surface temperature [44] and the increase in a 10-day running mean of daily mean temperatures for the actual sites during spring.

The meteorological data used in our study (i.e. the grid data and observations from the weather stations) should be considered as a fairly rough estimate for the local micro climate found near or at the ground surface in the habitat of the ticks. Tick development depends on the combination of RH and temperature, and these combined factors of RH and temperature had a highly significant effect on the moulting of engorged larvae into nymphs and engorged nymphs into adults. Previous studies have shown that SD affects tick dynamics and thus tick numbers, while RH on its own influences the virus replication level in the ticks [64]. Gilbert found that there was a strong negative effect of altitude on questing tick abundance, and deduced that the effect of altitude was probably due to temperature and humidity, e.g. with SD playing an important role [65].

It is crucial to study the microclimatic conditions in particular RH and SD (Figures 2 and 3) because the tick phenology, and therefore the degree of co-feeding transmission could be influenced by SD, as well as TBEV replication within the tick body possibly being influenced by RH [45,64,66,67]. Studies have shown that RH has an influence on the maintenance of the TBEV within ticks [64,68]. Experimental studies show that low humidity promotes the disappearance of the virus from the tick body [68], and that higher humidity affects the multiplication of the virus in ticks and influences the degree of overall virus infection rate in the tick population [64]. An increased SD causes a decreased tick density (or decreased availability) and limits questing duration, and that SD is a key factor that affects not only questing nymph abundance, but also the seasonal pattern of nymph questing activity [15,66,69]. Larvae are more sensitive to even smaller changes in SD than nymphs [15,70], and once SD becomes so high that it limits tick questing, the TBEV transmission will be lower [13]. High SD also affects tick survival [66] since if high SD prevail, ticks will eventually use up their fixed energy reserves and die. Thus long-lasting high SD might limit the development of tick populations and affect tick abundance. It is not primarily the density of ticks (ticks per square meter or ticks per volume) that is reduced when the density of water molecules in the air at ground level decreases. Instead, the activity of the ticks, which to the collector gives the impression that the tick density has diminished – in reality it is the availability (the product of tick density and tick activity) that is reduced by the increasing “deficit” of water in the air [52]. In our study, we found that the site with the lowest mean RH also had the lowest EPP, whereas the site with the highest mean RH had the correspondingly highest EPP, which is in accordance with Naumov and Danielova’s findings [64,68]. The literature indicates that SD acts primarily on the TBEV transmission pathway by reducing the degree of co-feeding, while RH seems to directly affect the level of TBEV replication within the tick population. Our results suggest that microclimatic conditions, particularly RH and SD, are of major importance locally, and are in line with previous findings [13,15,45]. In order to improve the representativeness of the climate data, it is necessary to perform measurements of temperature and humidity at the actual site and at sites where *I. ricinus* is abundant but where TBEV seems to be absent, which will be considered in future studies.

## Conclusions

The limited previous information on the dispersion of TBEV in southern Norway makes this study of TBEV in ticks of major importance in relation to increasing the knowledge of TBEV risk areas. The seven foci of TBEV in this study had an estimated overall prevalence rate in pools of ticks combined of 0.53%, with EPP point estimates ranging from 0.11%–1.22% for the various locations. We have compared two methods for pooled prevalence estimation, MIR and EPP. The method of choice is EPP, since it provides estimated prevalences with confidence intervals opposed to MIR. Our study confirms the existence of TBEV endemic foci in Norway and demonstrates the necessity of further studies on TBEV in nymphs as they are most abundant and responsible for the majority of tick bites.

Our results indicate that the location with the highest estimated prevalence had the correspondingly highest relative humidity and lowest saturation deficit, while the location with the lowest estimated prevalence had the correspondingly lowest relative humidity and highest saturation deficit. These findings emphasise the possible importance of microclimatic conditions regarding the TBEV prevalence in ticks.

## Abbreviations

TBE, Tick-Borne Encephalitis; TBEV, Tick-Borne Encephalitis Virus; EPP, Estimates of Pooled Prevalence and confidence limits; MIR, Minimum Infection Rate; MSIS, Meldingssystem for smittsomme sykdommer, In english the Norwegian Surveillance System for Communicable Diseases; LST, Land Surface Temperature; RH, Relative Humidity; SD, Saturation Deficit; Ct, Cycle threshold; RT-PCR, Reverse transcription polymerase chain reaction.

## Competing interests

The authors declare that they have no competing interests.

## Authors’ contributions

ÅA made substantial contributions to conception, design and coordination of the study, carried out the molecular genetic studies, statistical analysis of the pooled prevalence and drafted the manuscript and wrote substantial parts of the final manuscript. SJ made substantial contributions to conception and design, collected ticks, performed the pooled sampling and pooled prevalence estimations, interpretation of prevalence and climatic data with confidence limits and the statistical analyses of the climatic data, drafted and wrote substantial parts of the final manuscript. PC made substantial contributions to conception, acquisition and tick analysis and was involved in drafting the manuscript. SD made substantial contributions to conception and design of the study, statistical analysis of the pooled prevalence and drafted the manuscript. TT designed the real-time PCR method and drafted the manuscript. KI and HOH contributed with the extrapolation, calculation and discussion of climatic data. HV contributed with statistical discussion, presentation and discussion of climatic data and drafting the manuscript. PO made substantial contributions to conception and design, collected ticks, and drafted the manuscript. GÅ participated in the design of the study, drafting the manuscript and revising it critically for important intellectual content. KV made substantial contributions to conception and design, gave guidance on the molecular genetic studies, drafted the manuscript and gave final approval of the version to be published. All authors read and approved the final manuscript.

## Supplementary Material

Additional file 1 Appendix Information about the distribution of TBEV in Norway. Click here for file

Additional file 2 **Figure S1.** TBEV estimated pooled prevalence with 95% CI for the seven locations. The locations S1 (blue) and S7 (purple) are significantly different from the location S5 (red). Click here for file

Additional file 3 **Table S1.** Relative risk (RR) for all sites compared to S1 with confidence interval (CI) using likelihood ratio tests. Click here for file
