# Supplementary material for: Prevalence of tick borne encephalitis virus in tick nymphs in relation to climatic factors on the southern coast of Norway
Source: Parasit Vectors. 2012 Aug 22;5:177. doi: 10.1186/1756-3305-5-177 (PMC3497858; doi:10.1186/1756-3305-5-177)
Supplement: Additional file 1 — Appendix Information about the distribution of TBEV in Norway. [file 1756-3305-5-177-S1.docx]

**Appendix**

**Information about the distribution of TBEV in Norway**

In Norway, 75 human TBE cases have been reported between 1998-2011 along the coast of southern Norway (counties Vest- and Aust-Agder, Telemark and Vestfold, see figure 1 in the manuscript) [1], A study conducted in Arendal (Aust-Agder) has shown that 16.4% of 317 dogs had antibodies against TBEV [2].

Two studies performed in the 1980s reported a few cases of Louping ill-like disease in sheep from south and south-western Norway (Farsund in Vest-Agder and Etne in Hordaland [3,4]. The virus was isolated from some of the cases and characterized as LIV indistinguishable from Scottish strains [5].

Results from studies performed in the past are indicative of that viruses similar to TBEV and LIV could circulate quite far north on the western coast of Norway. A serological study on cattle from Farsund to Ørskog (Møre and Romsdal at the north-western coast of Norway) showed that 14 of 81 animals were TBEV seropositive [6]. In another study performed in the counties of Hordaland, Sogn and Fjordane and Møre and Romsdal, 19.6% of 341 human patients were seropositive in a screening for TBEV[7].

In a seroprevalence study performed by the Norwegian Veterinary Institute, the Norwegian Institute of Public Health and Moredun Research Institute, Scotland, UK antibodies against LIV and TBEV were detected in wild cervids in Farsund, whereas in Molde (Møre and Romsdal) antibodies were only detected against TBEV (**'Bjoernar Ytrehus unpublished observations'**).

These results indicate that TBEV and LIV may co-circulate in southern Norway and that viruses antigenetically similar to TBEV may be found in north-western Norway. The climatic condition in the north-western part of Norway is regarded as suboptimal from what is established from published literature as characteristics for TBEV locations. The current finding of TBEV seropositive deer might indicate that the virus exist in small niches where microclimate is favourable along with other factors which support TBEV transmission, even though the general area is regarded as suboptimal for TBEV establishment.

**References**

1. Norwegian Institute of Public Health: **Norwegian Surveillance System for Communicable Diseases (MSIS).** 2012, [*http://www.msis.no*](http://www.msis.no).

2. Csángó PA, Blakstad E, Kirtz GC, Pedersen JE, Czettel B: **Tick-borne encephalitis in southern Norway.** *Emerg Infect Dis* 2004, **10:**533-534.

3. Ulvund M, Vik T, Krogsrud J: **[Louping-ill (tick-borne encephalitis) in sheep in Norway].** *Norsk veterinærtidskrift* 1983, **95:**639-641.

4. Ulvund M: **Louping-ill (Tick-borne encephalitis) hos sau på Vestlandet.** *Småfenytt* 1987, **7:**99-106.

5. Gao GF, Jiang WR, Hussain MH, Venugopal K, Gritsun TS, Reid HW, Gould EA: **Sequencing and antigenic studies of a Norwegian virus isolated from encephalomyelitic sheep confirm the existence of louping ill virus outside Great Britain and Ireland.** *J Gen Virol* 1993, **74 ( Pt 1):**109-114.

6. Traavik T: **Serological investigations indicating the existence of tick-borne encephalitis virus foci along the Norwegian coast.** *Acta Pathol Microbiol Scand B Microbiol Immunol* 1973, **81:**138-142.

7. Traavik T: **Antibodies to tick-borne encephalitis virus in human sera from the western coast of Norway.** *Acta Pathol Microbiol Scand B* 1979, **87B:**9-13.
