# Supplementary figures and images for: Prevalence of tick borne encephalitis virus in tick nymphs in relation to climatic factors on the southern coast of Norway
Source: Parasit Vectors. 2012 Aug 22;5:177. doi: 10.1186/1756-3305-5-177 (PMC3497858; doi:10.1186/1756-3305-5-177)

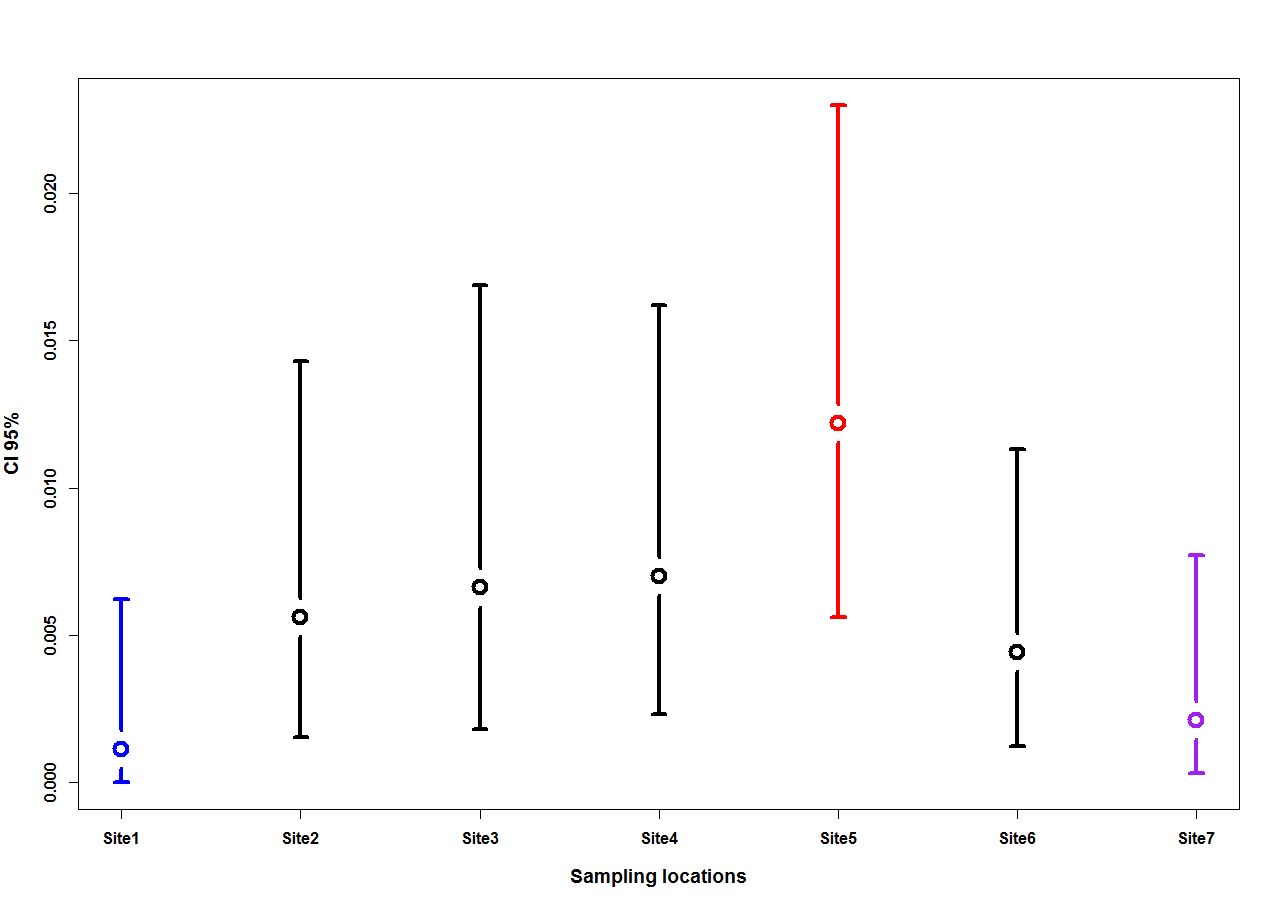

Supplement: Additional file 2 — Figure S1. TBEV estimated pooled prevalence with 95% CI for the seven locations. The locations S1 (blue) and S7 (purple) are significantly different from the location S5 (red). [file 1756-3305-5-177-S2.tiff]
