# Supplementary material for: Prevalence of tick borne encephalitis virus in tick nymphs in relation to climatic factors on the southern coast of Norway
Source: Parasit Vectors. 2012 Aug 22;5:177. doi: 10.1186/1756-3305-5-177 (PMC3497858; doi:10.1186/1756-3305-5-177)
Supplement: Additional file 3 — Table S1. Relative risk (RR) for all sites compared to S1 with confidence interval (CI) using likelihood ratio tests. [file 1756-3305-5-177-S3.docx]

| Table S1: Relative risk (RR) for all sites compared to S1 with confidence interval (CI) using likelihood ratio tests | | | | | | | |
| --- | --- | --- | --- | --- | --- | --- | --- |
| Sites compared | RR | CI | Statistics |  |  |  |  |
| S2 vs S1: | 4.93 | 0.55 - 44.1 | P = 0.15 |  |  |  |  |
| S3 vs S1: | 5.80 | 0.65 - 52.0 | P = 0.12 |  |  |  |  |
| S4 vs S1: | 6.08 | 0.71 - 52.1 | P = 0.10 |  |  |  |  |
| S5 vs S1: | 10.38 | 1.32 - 82.0 | P = 0.026 |  |  |  |  |
| S6 vs S1: | 3.91 | 0.44 - 35.0 | P = 0.22 |  |  |  |  |
| S7 vs S1: | 1.91 | 0.17 - 21.1 | P = 0.60 |  |  |  |  |
